# Single nucleus RNA sequencing and spatial transcriptomics reveal unique functionalities of gray matter versus white matter oligodendrocytes in aging and Alzheimer’s Disease

**DOI:** 10.64898/2026.08.29.747923

**Published:** 2026-09-02

**Authors:** Joseph P. Voth, Javier A. Ramos Benitez, Angela M. Wilson, Scott R. Kennedy, Mamatha Damodarasamy, Amanda Kirkland, Jeremy A. Miller, Caitlin S. Latimer, Emily Ragaglia, Amber Nolan, Daniel D. Child, Lisa M. Keene, Cindy L. Reichel, Brendan F. Kohrn, Larry S. Sherman, Stephen A. Back, C. Dirk Keene

## Abstract

Oligodendrocyte (OL) dysfunction and white-matter (WM) vulnerability are increasingly recognized as important aspects of aging and Alzheimer’s Disease (AD), yet human WM–focused, cellular-resolution transcriptomic data remain limited. Here, we profiled prefrontal WM from 48 brain donors spanning young adulthood and late-life with low versus high AD Neuropathologic Change (ADNC) using single-nucleus RNA sequencing followed by spatial transcriptomics (CosMx) in a subset of matched donors. We integrated aged WM OLs with a reference dorsolateral prefrontal cortex grey-matter (GM) OL dataset (SEA-AD) to define region- and pathology-associated OL programs. Across modalities, GM OLs exhibited a robust synapse/neurotransmitter-associated transcriptional signature relative to WM OLs, whereas this program was reduced with aging and attenuated in high ADNC GM. In contrast, WM OLs showed stronger immune-associated programs with aging and further enhancement in high ADNC, including cytokine/chemokine signaling and antigen presentation–related pathways. High ADNC WM OLs also displayed amplified proteostasis and stress-adaptation signatures, including selective upregulation of chaperone/heat shock genes and ferritin subunits, consistent with increased protein-folding demand and altered iron handling. To resolve OL state organization beyond static differential expression, we annotated OL sub-states using marker panels and inferred pseudotime-guided directional state-to-state flows within each tissue/condition stratum. This analysis identified a conserved newly formed differentiating (NFOL)/differentiating → lipid remodeling (APOE/ABCA1/LPL+) → Stress/ISR-reactive architecture, with a pronounced expansion of the Stress/ISR-reactive compartment and altered transition-associated pathway enrichment in high ADNC WM. Together, these data define WM-specific OL programs linked to aging and ADNC and nominate a stress/immune-enriched OL state landscape consistent with a putative senescence-like phenotype in diseased WM.

## Full Text Availability

The license terms selected by the author(s) for this preprint version do not permit archiving in PMC. The full text is available from the preprint server.

